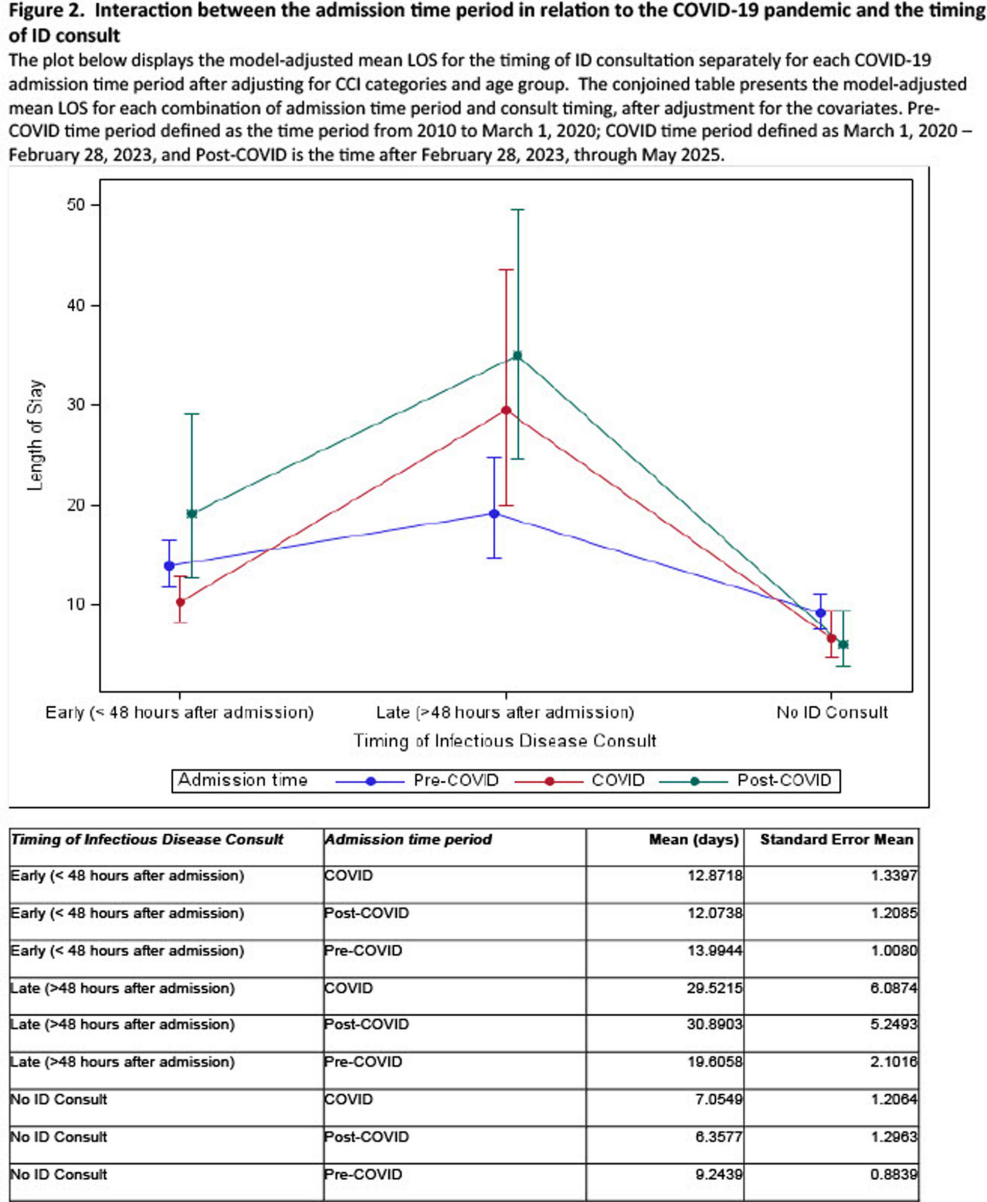# 362 Occurrence of Acinetobacter, Pseudomonas, and Klebsiella in patient room sinks and drains of a new hospital

**DOI:** 10.1017/ash.2026.10698

**Published:** 2026-06-23

**Authors:** Subhadra Mandadi, Elizabeth Lyden, Alyssa Lawrence, Nicole Arens, Matthew Anderson, Kevan English, Evangeline Green, Jason Payne, Jasmine Marcelin

**Affiliations:** 1 University of Nebraska Medical Center; 2 University of Nebraska Medical Center College of Medicine; 3 College of Medicine, University of Nebraska Medical Center, Omaha

## Abstract

**Background:** The indications for the use of cardiac and endovascular implants have expanded worldwide. With increased utilization, there has been a proportional increase in implant-related complications, including infections. Epidemiological factors influencing patient outcomes in cardiac and endovascular implant infections (CEVII) are poorly characterized. We aimed to evaluate the epidemiology of CEVII and further, how healthcare utilization (HCU) patterns and the timing of infectious disease (ID) consultation influence hospital length of stay, a known surrogate of patient morbidity and quality of care. **Methods:** This retrospective study included patients admitted between 2010 and May 2025 with a CEVII encounter ICD-10 diagnosis of T82.7XXA, excluding deaths. For patients with multiple admissions, only the first encounter was analyzed. Descriptive statistics summarized patient characteristics, and we evaluated associations with the timing of ID consultation, HCU, and LOS. LOS modeling used generalized linear mixed models with a negative binomial distribution and log link, including categorical predictors and their interactions; significant interactions indicated effect modification. Model-adjusted mean LOS and 95% confidence intervals were obtained by exponentiating model coefficients. Analyses were performed in SAS 9.4, with p? **Results:** Among 763 patients with CEVII (1,168 admissions from 2013–May 2025), most were male (62%), White (76%), and Medicare beneficiaries (63%), with a median age of 61 years, median LOS of 8 days, and median Charlson Comorbidity Index (CCI) of 5.5. Early ID consult (<48 hrs.) occurred in 58.1% of cases, while 24% had no ID consult. Age and CCI were independent predictors of LOS, with no significant interaction with HCU. After adjustment, insurance type and the timing of ID consult significantly influenced LOS (p=0.0067), with late ID consults being associated with longer stays among Medicare and private insurance beneficiaries. Timing of ID consultation also interacted with admission period (pre-COVID, COVID, and post-COVID), with early ID consultationconsistently reducing LOS, while late consults resulted in the longest LOS, a difference amplified during the pandemic. **Conclusion:** Older patients with suspected CEVII who have high HCU and severe CCI experience longer LOS, underscoring the need for proactive care bundles to reduce excess stay and associated burden. Early ID consultation is critical, especially for Medicare and privately insured patients, where delays can lead to LOS penalties. Strengthening coordinated workflows to prevent delayed ID consultations during system strains, such as pandemics, can help avoid unnecessary prolonged hospitalizations and their impacts.

**Figure:**
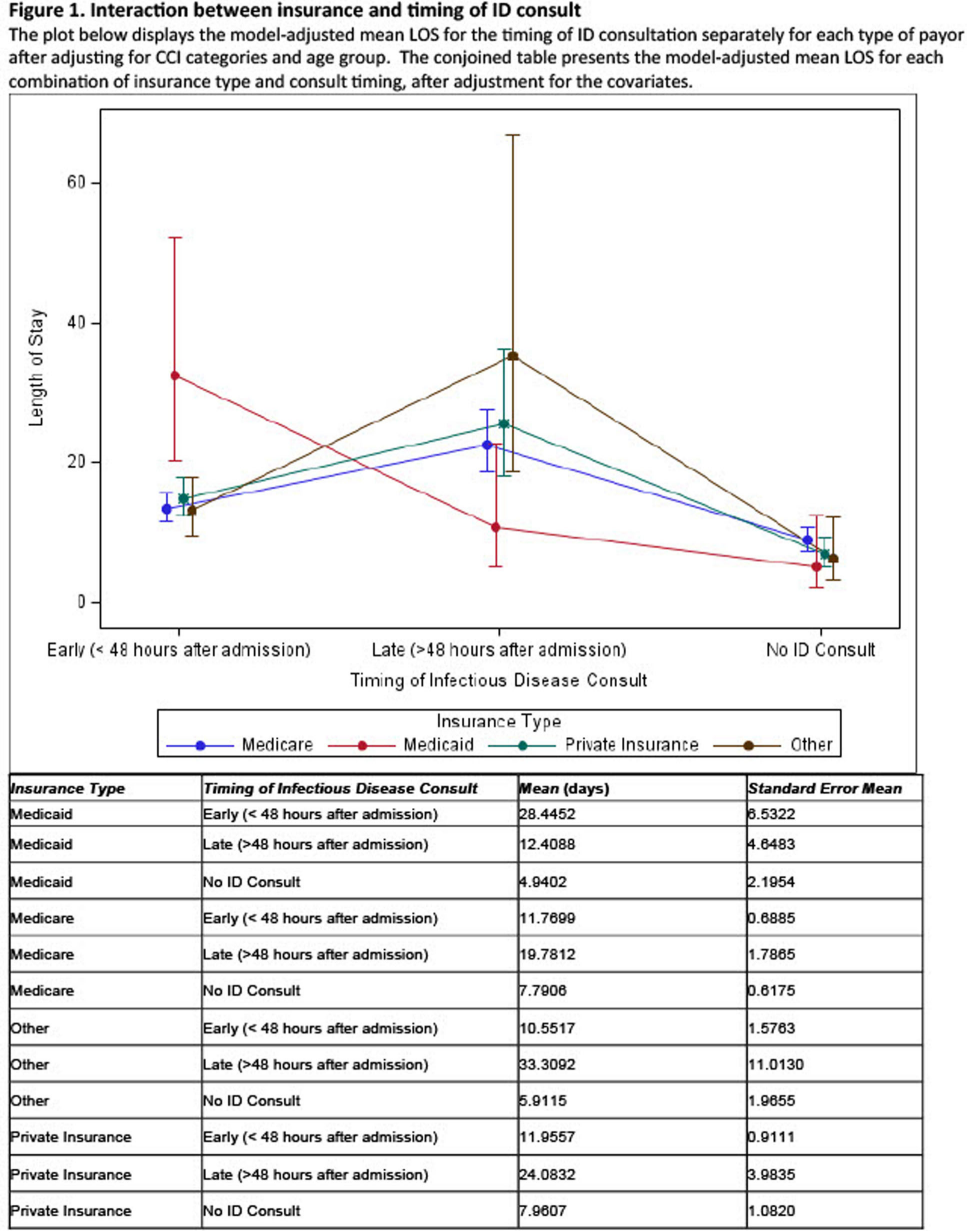

**Figure:**